# A Curious Case of Vanishing Hepatocellular Carcinoma (HCC)

**DOI:** 10.7759/cureus.46576

**Published:** 2023-10-06

**Authors:** Robert Kwei-Nsoro, Bashar Attar, Pius E Ojemolon, Eugene N Annor, Benjamin Go

**Affiliations:** 1 Internal Medicine, John H. Stroger, Jr. Hospital of Cook County, Chicago, USA; 2 Gastroenterology and Hepatology, Rush University Medical Center, Chicago, USA; 3 Gastroenterology and Hepatology, John H. Stroger, Jr. Hospital of Cook County, Chicago, USA; 4 Internal Medicine, University of Illinois, Chicago, USA; 5 Gastroenterology, John H. Stroger, Jr. Hospital of Cook County, Chicago, USA

**Keywords:** systemic inflammatory response, tumor hypoxia, alcohol related cirrhosis, spontaneous tumor regression, hepatocellular carcinoma (hcc)

## Abstract

Hepatocellular carcinoma (HCC) is the third most common cause of cancer-related mortality worldwide. Spontaneous regression of HCC is rare with few documented cases in literature. The mechanism of this phenomenon is unknown, but tumor hypoxia and systemic inflammatory response have been suggested as possible etiologies. This article aims to shed more light on this rare phenomenon and provides an opportunity to review the proposed pathophysiology of spontaneous HCC regression. In this case report, we describe an interesting case of a 39-year-old male with HCC who underwent spontaneous regression.

## Introduction

Hepatocellular carcinoma (HCC) is a primary liver tumor that usually occurs in the setting of cirrhosis. It is the third leading cause of cancer-related deaths in the world, according to the World Health Organization GLOBOCAN database, and the most rapidly growing cause of cancer deaths in the United States [1,2]. The therapeutic options for HCC include resection, radiofrequency ablation, transarterial chemoembolization (TACE), and systemic chemotherapy. HCC can rarely undergo spontaneous regression; we describe an interesting case of a 39-year-old male with HCC that underwent spontaneous regression.

## Case presentation

**Figure 1:**
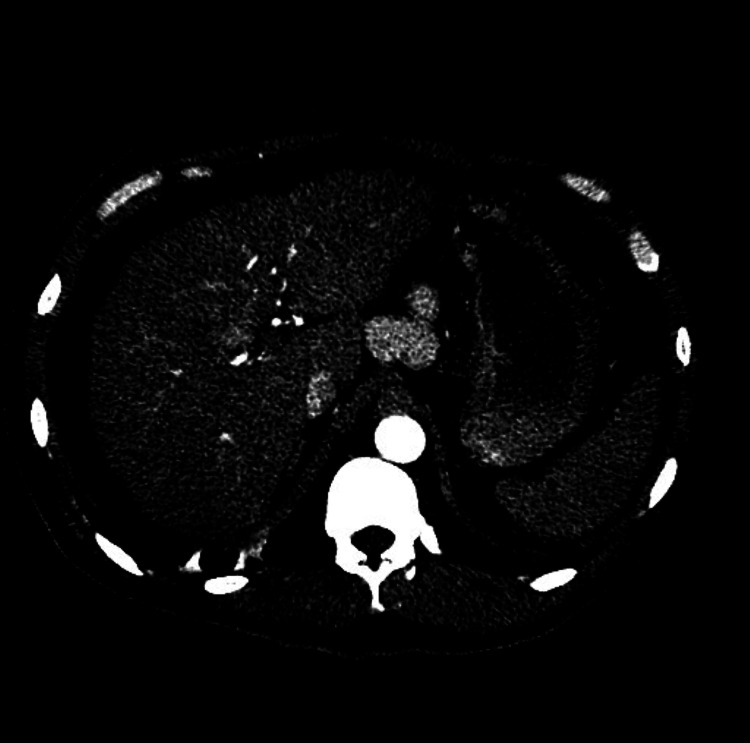
Triple phase CT of the abdomen showing a liver mass in segment VIII with mild arterial enhancement

**Figure 2:**
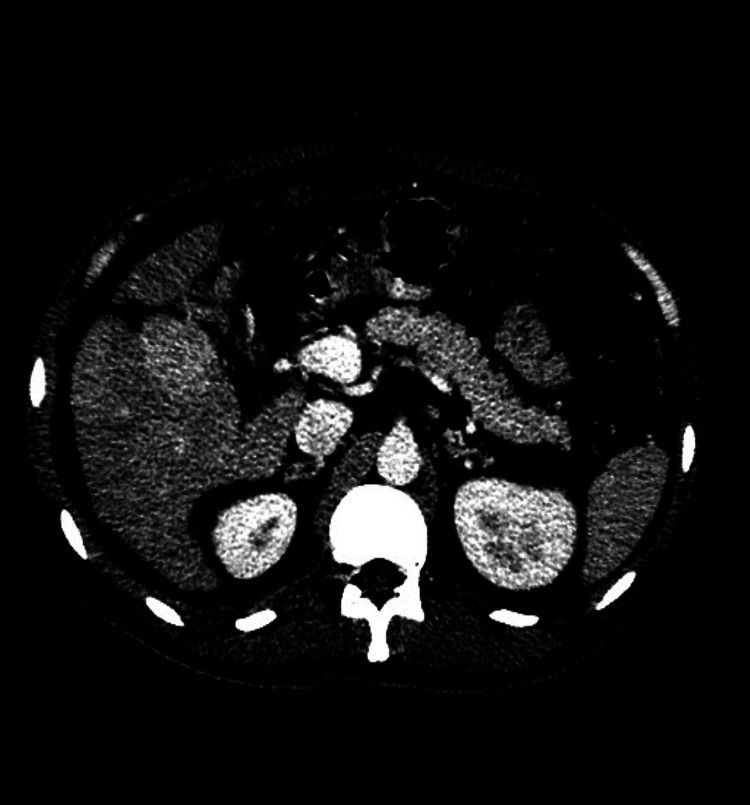
Enhancing mass in segment VIII of the liver during the porto-venous phase

**Figure 3:**
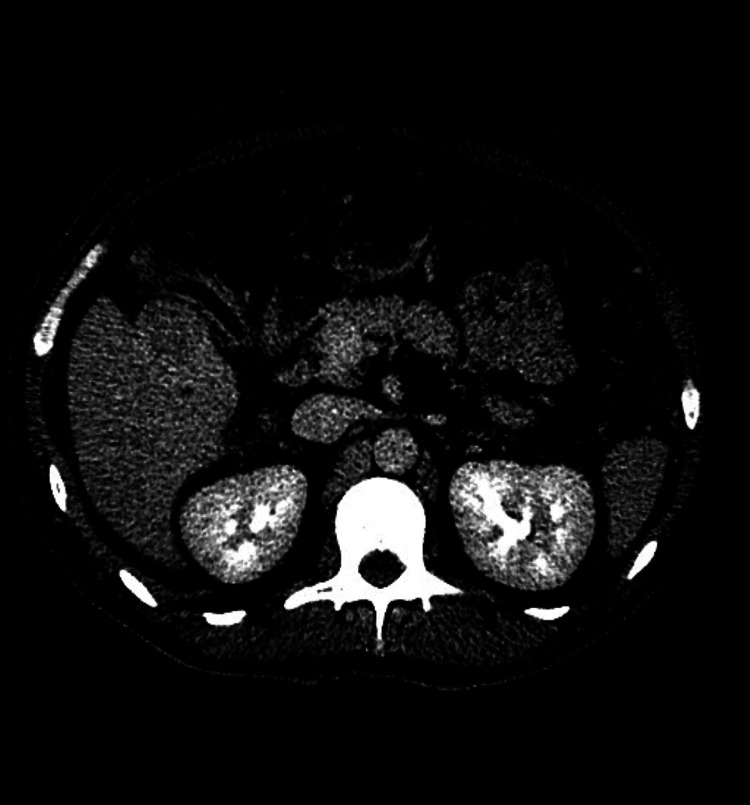
Rapid washout of liver mass on delayed phase imaging

**Figure 4:**
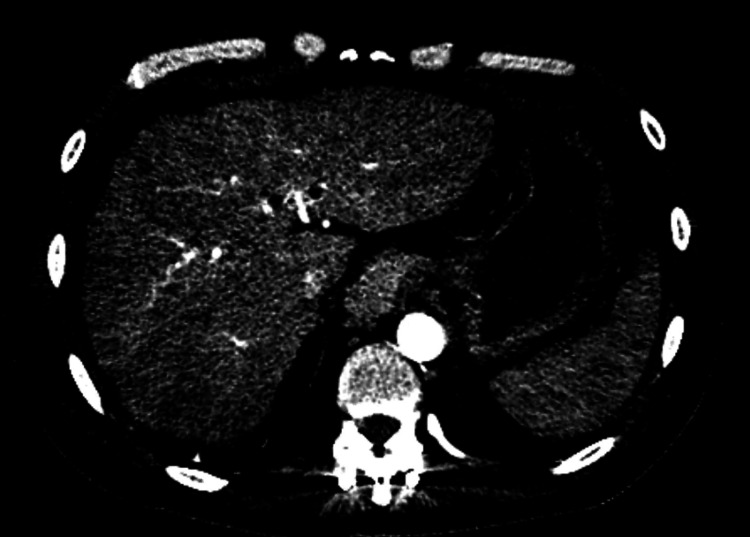
Cirrhotic morphology of the liver without arterially enhancing masses previously noted

A 39-year-old male with alcohol-associated cirrhosis (Child C, MELD (Model for End-Stage Liver Disease) 23) underwent evaluation of abdominal pain in the emergency room with computed tomography (CT) with contrast. The CT revealed cirrhotic morphology of the liver with a 4 cm hyper-enhancing mass in the right hepatic lobe. Triple-phase CT done for further evaluation of the hepatic mass noted on the single-phase study showed an arterially enhancing mass in segment VIII, which measured at least 3.6 cm in axial dimension and washes out on delayed phase CT; this was classified as LI-RADS 5 lesion (imaging features consistent with HCC, Figures 1-3). There was an additional arterially enhancing 1.5 cm nodule in segment VIII with probable washout, suspicious for hepatocellular carcinoma, LI-RADS 4 lesion. The third lesion identified was an arterially-enhancing subcentimeter nodule in the anterior right hepatic lobe, classified as LI-RADS 3. His alpha-fetoprotein level at the time was 8.84 (normal: <10ng/mL). The patient was deemed not a candidate for any treatment based on his Child-Pugh score so he was offered supportive care. He was lost to follow-up till about three years after his initial presentation when he was again admitted for evaluation of abdominal pain. During the prior three years, he abstained from alcohol consumption evidenced by improvement in his MELD score (Child B, MELD 17). He also got no specific treatment for the HCC. Imaging (CT abdomen) done for evaluation of his abdominal pain did not show any arterially enhancing hepatic lesions previously identified. He subsequently got a triple-phase CT which showed tiny hypodense hepatic lesions but no arterially enhancing lesions (Figure 4). His alpha-fetoprotein level remained low at 6.12 ng/mL. The patient remains HCC-free four years after his initial diagnosis following complete regression of the lesion without any treatment.

## Discussion

Spontaneous regression of HCC is a rare phenomenon, and there have been a few documented cases of spontaneous HCC regression in the United States [3]. Spontaneous regression has mostly been described in males in East Asian countries [4]. It was first described by Johnson and colleagues in 1972 [5]. They described a three-year-old girl who had developed biopsy-proven HCC while on chronic androgen-anabolic steroid treatment for aplastic anemia. A year after her diagnosis, she was admitted for treatment of *Staphylococcus aureus* sepsis at which time her imaging demonstrated near-complete resolution of her HCC [5]. The true incidence of spontaneous HCC regression is difficult to determine. However, a meta-analysis of ten randomized control trials reported a regression incidence rate of 0.41% [6]. The mechanism by which spontaneous regression occurs is unclear, but two mechanisms have been proposed: tumor hypoxia and systemic inflammatory response.

Tumor hypoxia as a mechanism is inherently appealing as it simulates the conditions created by certain established treatment modalities such as TACE and sorafenib. There are some documented cases of spontaneous regression where patients were noted to have direct ischemic insult either through thrombosis of the hepatic artery or portal vein or profound systemic hypoperfusion from massive variceal hemorrhage [7-9]. TACE reduces blood supply to the tumor by occluding the vessel and artificially inducing a similar condition as has been described in some case reports of spontaneous regression after spontaneous arterial thrombosis [10]. Sorafenib due to its effect on vascular endothelial growth factor is thought to inhibit neovascularization of the malignancy and the results appear to mirror the conditions described in the tumor hypoxia mechanism in spontaneous regression [11].

There have been some reports of spontaneous regression of metastatic disease which suggests a systemic process may possibly be involved in spontaneous regression [12-14]. Abstinence from alcohol and smoking, prolonged fever, vitamin K administration, and certain herbal products have been described to induce a systemic inflammatory response [15-18]. Several reports have documented elevated cytokine levels in patients with spontaneous regression suggesting the presence of a systemic inflammatory response [19,20]. Interleukin 18, tumor necrosis factor-alpha, and interferon-gamma are some of the inflammatory cytokines that are elevated in patients with spontaneous regression [19,20]. The reported immune responses have formed the basis of some newer immune-based systemic therapies such as atezolizumab and durvalumab.

The characteristics of the HCC in this present case were large multifocal lesions in a young adult with significant alcohol intake. The exact mechanism for the spontaneous regression observed here is unclear but it is possibly related to his abstinence from alcohol and/or a spontaneous venous or arterial thrombosis that went undiagnosed when the patient was lost to follow-up.

## Conclusions

With regard to our patient, alcohol cessation, which has been shown to induce a form of systemic inflammatory response, might have contributed to the spontaneous regression of his HCC. The data reviewed here shows that tumor hypoxia and systemic inflammatory response are important components of spontaneous regression. The true mechanism of spontaneous HCC is likely complex and multifactorial. It is entirely plausible that these two factors described likely have a synergistic effect on spontaneous regression. Analysis of the immunological reactions involved in the spontaneous regression of HCC has serious implications for the development of newer forms of immune-based therapies. It is, therefore, clear that the accumulation of more literature on the spontaneous regression of HCC is necessary in this active area of research.
